# Calcium-binding protein 7 expressed in muscle negatively regulates age-related degeneration of neuromuscular junctions in mice

**DOI:** 10.1016/j.isci.2024.108997

**Published:** 2024-01-26

**Authors:** Takahiro Eguchi, Tohru Tezuka, Yuji Watanabe, Akane Inoue-Yamauchi, Hiroshi Sagara, Manabu Ozawa, Yuji Yamanashi

**Affiliations:** 1 Division of Genetics, The Institute of Medical Science, The University of Tokyo, Tokyo 108-8639, Japan; 2 Medical Proteomics Laboratory, The Institute of Medical Science, The University of Tokyo, Tokyo 108-8639, Japan; 3 Laboratory of Reproductive Systems Biology, The Institute of Medical Science, The University of Tokyo, Tokyo 108-8639, Japan; 4 Core Laboratory for Developing Advanced Animal Models, The Institute of Medical Science, The University of Tokyo, Tokyo 108-8639, Japan

**Keywords:** Physiology, Molecular biology, Neuroscience

## Abstract

The neuromuscular junction (NMJ) forms centrally in myotubes and, as the only synapse between motor neuron and myotube, are indispensable for motor activity. The midmuscle formation of NMJs, including midmuscle-restricted expression of NMJ-related genes, is governed by the muscle-specific kinase (MuSK). However, mechanisms underlying MuSK-mediated signaling are unclear. Here, we find that the *Calcium-binding protein 7* (*Cabp7*) gene shows midmuscle-restricted expression, and muscle-specific depletion of *Cabp7* in mice accelerated age-related NMJ degeneration, muscle weakness/atrophy, and motor dysfunction. Surprisingly, forced expression in muscle of CIP, an inhibitory peptide of the negative regulator of NMJ formation cyclin-dependent kinase 5 (Cdk5), restored NMJ integrity and muscle strength, and healed muscle atrophy in muscle-specific Cabp7-deficient mice, which showed increased muscle expression of the Cdk5 activator p25. These findings together demonstrate that MuSK-mediated signaling induces muscle expression of Cabp7, which suppresses age-related NMJ degeneration likely by attenuating p25 expression, providing insights into prophylactic/therapeutic intervention against age-related motor dysfunction.

## Highlights

• *Cabp7* shows midmuscle expression close to NMJs and diminishes the Cdk5 activator p25
• Cabp7 loss in muscle promotes age-related NMJ degeneration and CMAP decrement in mice
• Cabp7 loss in muscle harms motor function, muscle strength/mass, and lifespan in mice
• Cdk5 inhibitor CIP treatment heals NMJ and motor defects in mice lacking muscle Cabp7

## Graphical abstract

**Figure:**
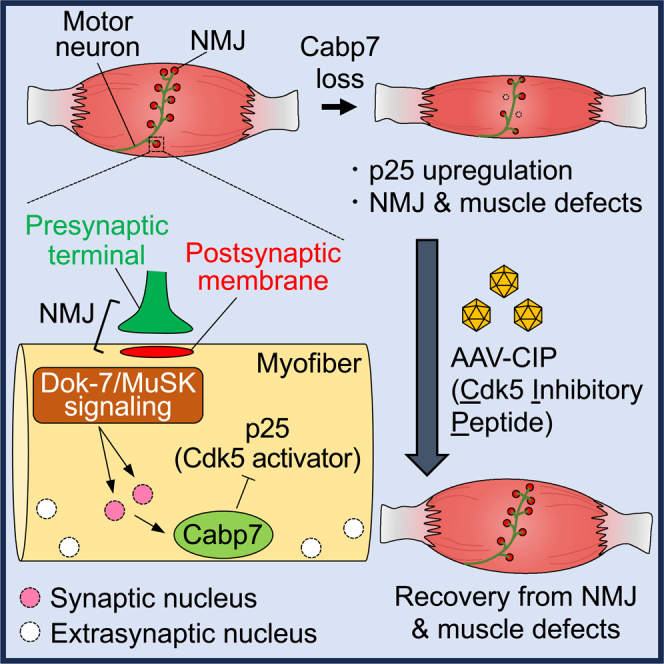

## Introduction

Motor nerves control skeletal muscle contraction via the neuromuscular junction (NMJ), the only synapse between motor neuron and myotube (including its mature form, a myofiber).^1^ Indeed, functional impairment of NMJs causes myasthenia, characterized by fatigable muscle weakness and atrophy.^1^ In mammals, the neurotransmitter acetylcholine (ACh) is synthesized by choline acetyltransferase (ChAT), transported from the neuronal cytoplasm into the synaptic vesicles by vesicular ACh transporter (VAChT), and released into the synaptic cleft between the nerve terminal and the muscle surface to bind and activate its ligand-gated ion channel receptors (AChRs) or otherwise to be degraded by acetylcholinesterase (AChE), which plays an essential role in muscle contraction or relaxation, respectively. In general, a single NMJ forms in the central region of each myotube and efficient NMJ transmission requires dense clustering of AChRs on the midmuscle, postsynaptic membrane.^1^ Rodent studies demonstrated age-related degeneration of NMJs, including NMJ denervation, which indicates impaired neuromuscular transmission with aging.^2^^,^^3^ Similarly, electrophysiological and muscle fiber-type studies together with gene and protein expression analyses using human skeletal muscle tissue demonstrated that age-related denervation at NMJs contributes to sarcopenia: the pathogenic loss of muscle mass and strength in the elderly.^4^^,^^5^^,^^6^^,^^7^^,^^8^ In addition, other electrophysiological studies further demonstrated a strong correlation between neuromuscular transmission decline and skeletal muscle atrophy during aging in both rats and humans.^9^ Thus, it is widely accepted that the age-related motor impairment is caused, at least in part, by functional decline of the NMJ.

Formation and maintenance of NMJs in the central region of the skeletal muscle are orchestrated by the muscle-specific receptor tyrosine kinase MuSK.^1^ Before NMJ formation during embryogenesis, the transcripts and protein products of AChR subunit genes are expressed, and accumulate specifically in the central region of the myotube in a manner dependent on MuSK.^10^ A growing body of evidence demonstrates that in multi-nucleated myotubes, only a small number of nuclei adjacent to the postsynaptic membrane of NMJs, called “postsynaptic nuclei”, are responsible for the midmuscle-restricted transcription of AChR subunit and other NMJ-related genes, including those encoding AChE and MuSK.^11^^,^^12^^,^^13^^,^^14^ Interestingly, although the midmuscle-restricted expression of AChR subunit genes and AChR clustering in embryos requires MuSK and its essential, muscle-intrinsic activator Dok-7, the motor nerve-derived MuSK activator agrin was dispensable for midmuscle-specific regulation.^10^^,^^15^^,^^16^^,^^17^ However, upon motor innervation during embryogenesis, the neural agrin further activates MuSK by binding to MuSK’s co-receptor low-density lipoprotein receptor-related protein 4 (Lrp4),^18^^,^^19^ and stabilizes the postsynaptic AChR clusters by counteracting the ACh-mediated dispersal of AChR clusters.^1^ Indeed, although agrin-deficient, but not MuSK- or Dok-7-deficient, mice show AChR clustering before innervation, those clusters are dispersed by motor nerve-derived ACh upon subsequent motor innervation.^16^^,^^17^^,^^20^^,^^21^^,^^22^

The ACh-mediated AChR cluster dispersal is regulated, at least in part, by cyclin-dependent kinase 5 (Cdk5).^1^^,^^23^ Cholinergic stimulation of cultured myotubes activates the Ca^2+^-dependent protease calpain,^24^ which cleaves the Cdk5 activator p35 into the more potent activator p25,^23^ leading to hyperactivation of Cdk5 and dispersal of AChR clusters.^24^ Moreover, genetic deletion or pharmacological inhibition of Cdk5 leads to AChR cluster retention and improves neuromuscular synaptogenesis in agrin-deficient mice.^22^ Thus, NMJ formation is negatively regulated by ACh-mediated signaling via Cdk5. Interestingly, aberrantly enhanced neuromuscular transmission by overexpression of VAChT in the presynaptic motor nerve terminals in mice accelerates age-related degeneration of NMJs,^25^^,^^26^ implying that ACh-mediated signaling may also play a role in promoting age-related NMJ degeneration. However, it remains unclear whether Cdk5-mediated mechanisms are involved in age-related degeneration of NMJs.

Calcium-binding protein 7 (Cabp7, also known as calneuron II) is a calmodulin superfamily member having two cytosolic EF-hand motifs for Ca^2+^ binding and a C-terminal transmembrane domain,^27^ which is abundantly expressed in neurons of rat brain.^28^ In this study, we demonstrate that *Cabp7* transcripts accumulate specifically in the central region of muscle, and that forced expression of Dok-7 in skeletal muscle, which induces MuSK activation,^15^ enhanced the midmuscle-restricted expression of *Cabp7* gene. Furthermore, we also demonstrate that Cabp7 plays a key role in slowing age-related degeneration of NMJs, likely, at least in part, by downregulating the Cdk5 activator p25. Indeed, forced expression of the p25-derived Cdk5 inhibitor CIP (Cdk5 inhibitory peptide)^29^^,^^30^^,^^31^ in muscle restored NMJ integrity and muscle strength, and healed muscle atrophy in muscle-specific Cabp7-deficient mice. Together, our findings provide insights into age-related NMJ degeneration and motor dysfunction, and also into potential prophylactic and therapeutic intervention against such disorders.

## Results

### Forced expression of Dok-7 in skeletal muscle enhances the midmuscle-restricted expression of the *Cabp7* gene

**Figure 1:**
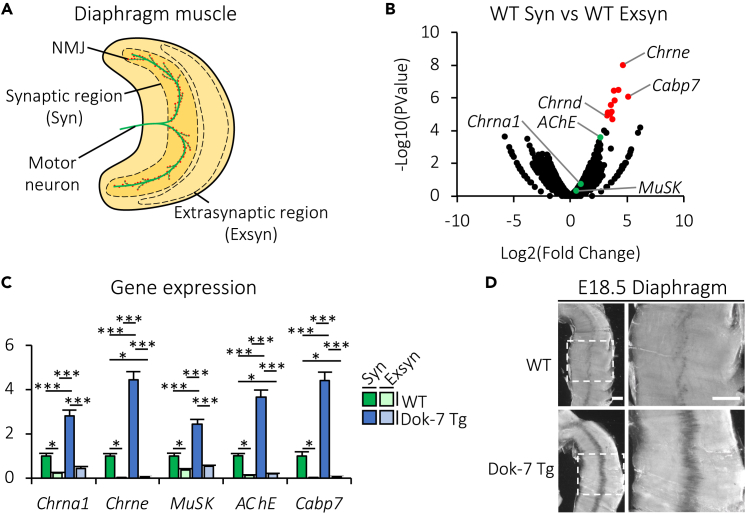
Forced expression of Dok-7 in skeletal muscle enhances midmuscle-restricted gene expression of *Cabp7* in mice (A) Diagram of mouse diaphragm muscle (red dots indicate postsynaptic AChR clusters of NMJs). Synaptic (Syn) and extrasynaptic (Exsyn) regions of diaphragm muscle are encircled with dotted lines. (B) RNA sequencing analysis of the synaptic and extrasynaptic regions of diaphragm muscles in WT mice at 3 months of age (n = 3 per group). Red dots indicate genes with false discovery rates (FDR) < 0.05, and green dots indicate *AChE*, *Chrna1*, or *MuSK*. (C) Quantified results for *Chrna1*, *Chrne*, *MuSK*, *AChE*, and *Cabp7* mRNA expression in synaptic (Syn) and extrasynaptic (Exsyn) regions of diaphragm muscles in WT and Dok-7 Tg mice at 3 months of age. The mean value of each gene expression level normalized to *Hprt* expression in the synaptic region of diaphragm muscle in WT mice were arbitrarily defined as 1. Error bars indicate mean ± SEM (n = 6 per group). Asterisks denote a significant statistical difference: ∗p < 0.05, ∗∗∗p < 0.001 by ANOVA followed by Tukey’s *post hoc* test. “N.S.” is not indicated for better visibility. (D) Representative images of diaphragm muscles of WT and Dok-7 Tg mice at E18.5 subjected to *in situ* hybridization with an antisense probe for *Cabp7*. The right panels represent magnified views of the insets in the left panels. Scale bars, 0.5 mm.

As mentioned previously, Dok-7 and MuSK together control not only AChR clustering but also transcription of AChR subunit and other NMJ-related genes in the central region of muscle, probably through restriction of transcription itself to postsynaptic nuclei.^11^^,^^12^^,^^13^^,^^14^^,^^15^^,^^16^^,^^17^ Indeed, we previously demonstrated that midmuscle expression of the AChR subunit gene *Chrna1* and *MuSK* transcripts are lost or enhanced in mouse embryos lacking the essential MuSK activator Dok-7 or overexpressing it specifically in skeletal muscle (Dok-7 transgenic (Tg) mice), respectively, compared with wild-type (WT) embryos.^15^ Thus, genes expressed specifically in the central, synaptic region of the muscle appear to be involved in the formation and/or maintenance of NMJs downstream of MuSK. To identify candidate genes required for the formation and/or maintenance of NMJs, we performed RNA sequencing analysis of the synaptic and extrasynaptic regions of diaphragm muscles in 3-month-old WT mice (Figure 1A). We confirmed significantly higher expression levels of the AChR subunit gene (*Chrne* and *Chrnd*) transcripts in the synaptic region, compared with the extrasynaptic region (Figure 1B), and found that *Cabp7* is the most upregulated among significantly upregulated genes in the synaptic region (red dots in Figure 1B), suggesting the possibility that the expression pattern of the *Cabp7* gene in skeletal muscle could be controlled by MuSK-mediated signaling. We thus examined the expression level of the *Cabp7* gene in synaptic and extrasynaptic regions of diaphragm muscles in WT and Dok-7 Tg mice at 3 months of age by reverse transcription quantitative PCR (Figure 1C). Like *Chrna1*, *Chrne*, *MuSK*, and *AChE*, the expression level of the *Cabp7* gene was significantly higher in synaptic than extrasynaptic regions in both WT and Dok-7 Tg mice, and was enhanced in the synaptic region in Dok-7 Tg mice compared with that in WT mice (Figure 1C). To examine the expression pattern of the *Cabp7* gene in muscle further, we performed whole-mount *in situ* hybridization on embryos of Dok-7 Tg and WT mice, and found that *Cabp7* transcripts were expressed specifically in the central region of the diaphragm muscle in both WT and Dok-7 Tg mice (Figure 1D). In addition, this specific expression of *Cabp7* transcripts was enhanced in Dok-7 Tg mice compared with that in WT mice (Figure 1D). Together, these results indicate that skeletal muscle-specific overexpression of Dok-7 enhances the midmuscle-restricted expression of *Cabp7* in mice, suggesting involvement of Cabp7, heretofore with no established *in vivo* role, in MuSK-mediated formation and/or maintenance of NMJs.

### Muscle-specific deletion of Cabp7 reduces motor function, muscle strength, and lifespan in mice

**Figure 2:**
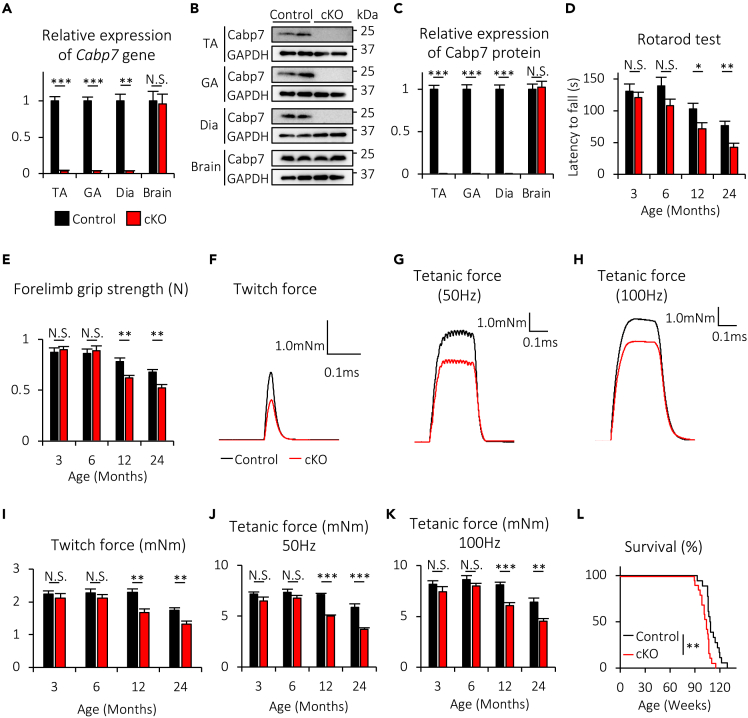
Cabp7 cKO mice show reductions in motor function, muscle strength, and life span (A) Quantification of *Cabp7* mRNA expression in the tibialis anterior (TA), gastrocnemius (GA), and diaphragm (Dia) muscles, and the brain of Cabp7 cKO and the control mice at 3 months of age (n = 5 per group). The mean value of *Cabp7* mRNA expression normalized to *Hprt* expression in the control mice was arbitrarily defined as 1. (B and C) Representative images of immunoblotting for Cabp7 and GAPDH (B) and quantitative data of Cabp7 protein expression normalized to GAPDH expression (C) in the TA, GA, and diaphragm muscles, and the brain of Cabp7 cKO and the control mice at 3 months of age (n = 4 per group). (D and E) Quantification of motor function and forelimb strength determined by rotarod test (D) and forelimb grip test (E), respectively, at the indicated ages (n = 12–16 per group). (F−H) Representative traces of twitch (F) and tetanic force at 50 Hz- (G) and 100 Hz-stimulations (H) at 12 months of age. (I−K) Quantification of twitch (I) and tetanic force at 50 Hz- (J) and 100 Hz-stimulations (K) at the indicated ages (n = 5–7 per group). (L) Kaplan-Meier survival curves of Cabp7 cKO and the control mice (n = 18–19 per group). Error bars indicate mean ± SEM. Asterisks denote a significant statistical difference: ∗p < 0.05, ∗∗p < 0.01, ∗∗∗p < 0.001 by unpaired Student’s *t* test (A, C, D, E, I, J, and K) and by log rank test (L). N.S., not significant. For detailed information on sample size, see Table S1.

To explore the role of Cabp7 in muscle, we generated *Cabp7*^*flox/flox*^ mice and crossed them with transgenic mice (*HSA-Cre* mice) expressing Cre recombinase under the control of the *human skeletal α-actin* (*HSA*) promoter, which is known to be muscle-specific (Figure S1).^32^ We then obtained *Cabp7*^*flox/flox*^;*HSA-Cre* male mice (Cabp7 conditional knockout (cKO) mice) and *Cabp7*^*flox/+*^;*HSA-Cre* male mice (“control mice” or “controls” hereafter), and confirmed that the expression levels of *Cabp7* mRNA and its protein products were greatly reduced in the tibialis anterior (TA), gastrocnemius (GA), and diaphragm (Dia) muscles, but not brain, of Cabp7 cKO mice, compared with those of the control mice at 3 months of age (Figures 2A–2C). Although there was no significant difference in body weight between Cabp7 cKO and the control mice at 3, 6, 12, and 24 months of age (Figure S2), Cabp7 cKO mice showed a significant reduction in motor function at 12 and 24, but not 3 or 6, months of age as determined by rotarod test (Figure 2D). Furthermore, Cabp7 cKO mice similarly showed reduced muscle strength at 12 and 24, but not 3 or 6, months of age in comparison with the controls, as determined by forelimb grip test (Figure 2E) and twitch and tetanic force tests of hindlimb muscle upon electrical stimulation (Figures 2F–2K). In addition, Cabp7 cKO mice have a lifespan about 8 weeks shorter than the control mice (Control: mean 111.6 ± 2.0 weeks; Cabp7 cKO: mean 103.5 ± 1.5 weeks) (Figure 2L). Together, these results indicate that skeletal muscle Cabp7 plays a key role in the maintenance of motor function and muscle strength during aging and is required ultimately for normal lifespan in mice.

### Muscle-specific deletion of Cabp7 accelerates age-related degeneration of NMJs

**Figure 3:**
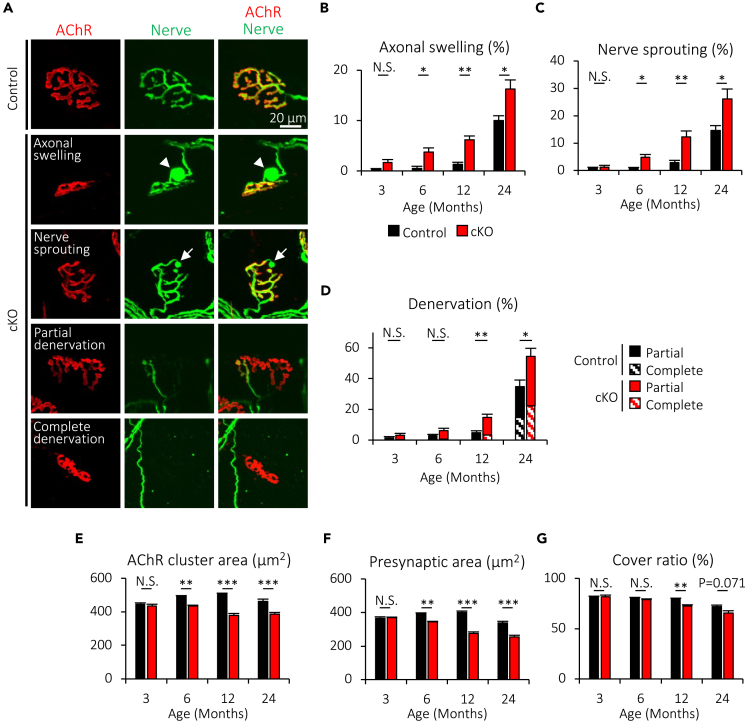
Cabp7 cKO mice show accelerated age-related NMJ degeneration (A) Representative images of NMJs in the TA muscles of Cabp7 cKO and the control mice at 12 months of age. The AChR clusters were visualized with α-bungarotoxin (red), and motor axons and presynaptic nerve terminals were stained with antibodies to neurofilament-L and synapsin-1 (green). Arrowheads or arrows indicate axonal swelling or nerve sprouting, respectively. (B−G) Quantification of the rate of axonal swelling (B), nerve sprouting (C), and denervation (D), the area of postsynaptic AChR clusters (E) and presynaptic motor nerve terminals (F), and the cover ratio of NMJs (G). Error bars indicate mean ± SEM (n = 4–6 per group). Asterisks denote a significant statistical difference: ∗p < 0.05, ∗∗p < 0.01, ∗∗∗p < 0.001 by unpaired Student’s *t* test. N.S., not significant. For detailed information on sample size, see Table S1.

The presynaptic motor nerve terminal is apposed to the postsynaptic membrane of the myotube, where in mammals AChRs densely cluster for efficient NMJ transmission.^1^ The pre- and post-synaptic specializations in mice show age-related degeneration including motor denervation, which is also reported in humans with aging.^2^^,^^3^^,^^4^^,^^8^ Furthermore, motor neurons often show sprouts beyond AChR clusters and/or axonal swelling adjacent to the postsynaptic sites of NMJs in aged rodents.^2^^,^^3^ To examine whether muscle-specific deletion of Cabp7 affects the morphology of NMJs, we examined NMJs in the TA muscle of Cabp7 cKO and the control mice using confocal microscopy (Figure 3A). As expected, the rates of axonal swelling and nerve sprouting gradually increased together with the denervation rates in the control mice (Figures 3B–3D). However, Cabp7 cKO mice showed significant increases in the rates of axonal swelling and nerve sprouting at 6, 12, and 24, but not 3, months of age, as compared with the controls (Figures 3B and 3C). Furthermore, Cabp7 cKO mice at 12 and 24, but not 3 or 6, months of age showed a significantly increased rate of denervation, in comparison with the controls (Figure 3D). In addition, Cabp7 cKO mice showed significant reductions in the areas of AChR clusters and presynaptic motor nerve terminals at 6, 12, and 24, but not 3 months of age, and in the cover ratio of presynaptic motor nerve terminals to postsynaptic AChR clusters at NMJs at 12 months of age, but not at other ages, while a nearly significant difference was shown at 24 months of age (p = 0.071) (Figures 3E–3G). These results indicate that age-related NMJ degeneration is accelerated as early as 6 months of age in Cabp7 cKO mice.

To clarify the role for Cabp7 in embryonic NMJ formation, we further investigated NMJ morphology at embryonic day 18.5 (E18.5) in mice, and found no significant changes in the areas of AChR clusters and presynaptic motor nerve terminals and the cover ratio in the diaphragm muscle of Cabp7 cKO embryos, as compared with those of the controls (Figures S3A‒S3D). These findings suggest that Cabp7 might not play an essential role in embryonic NMJ formation, even though Cabp7 cKO embryos showed residual *Cabp7* gene expression (about 20% of the control embryos) in the diaphragm muscle (Figure S3E), probably due to partial deletion of *Cabp7* genes at the embryonic stage of muscle development.

**Figure 4:**
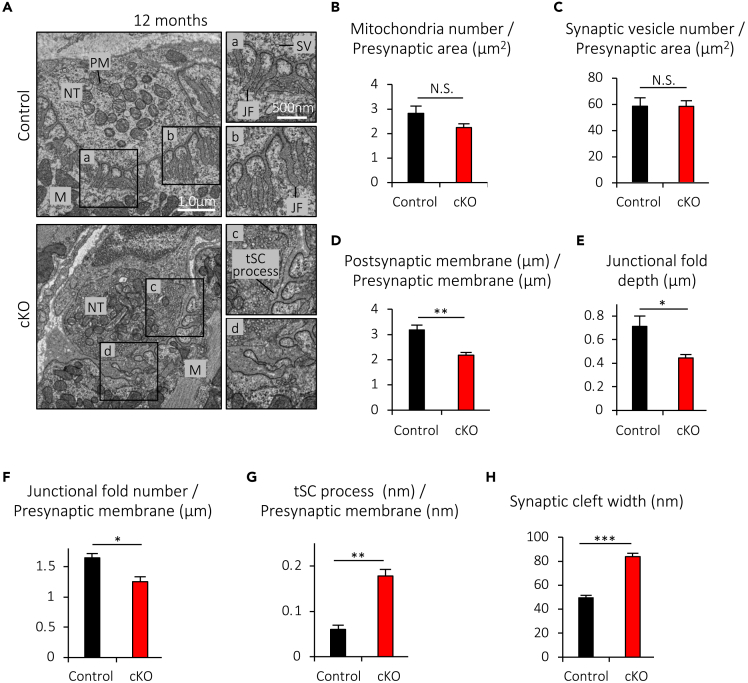
Muscle-specific deletion of Cabp7 induces ultrastructural degeneration of NMJs at 12 months of age (A) Representative transmission electron micrographs of NMJs in the diaphragm muscles of Cabp7 cKO and the control mice at 12 months of age. The right panels represent magnified views of the insets in the left panels (A−D). NT, nerve terminal; PM, presynaptic mitochondria; M, myotube (myofiber); JF, junctional fold; SV, synaptic vesicle; tSC, terminal Schwann cell. (B−H) Quantified results for the number of presynaptic mitochondria (B) and synaptic vesicles (C) in the indicated presynaptic area, the ratio of post-versus pre-synaptic membrane length of synaptic contacts (D), the depth of junctional folds (E), the density of junctional folds in the postsynaptic membrane of synaptic contacts (F), the total length of tSC processes in the synaptic clefts per nm presynaptic membrane length of synaptic contacts (G), and the synaptic cleft width between the pre- and post-synaptic membrane near the opening of junction folds (H). Error bars indicate mean ± SEM (n = 4 per group). Asterisks denote a significant statistical difference: ∗p < 0.05, ∗∗p < 0.01, ∗∗∗p < 0.001 by unpaired Student’s *t* test. N.S., not significant.

In addition to the structural features visualized by the confocal and fluorescent microscopy, the NMJ is also marked by ultrastructural features visualized by electron microscopy, including numerous invaginations of the postsynaptic muscle membrane, known as junctional folds, which are believed to be critical for efficient neuromuscular transmission at least in mammals.^1^^,^^33^ Furthermore, non-myelinating terminal Schwann cells (tSCs) cover the presynaptic nerve terminals at NMJs, and play an essential role in the maintenance of NMJs.^1^^,^^34^ With regard to aging, enhanced penetration of tSC processes into the synaptic clefts and degeneration of postsynaptic junctional folds together with increased synaptic cleft width in aged rodents have been reported.^35^^,^^36^ To examine whether muscle-specific deletion of Cabp7 enhances ultrastructural degeneration of NMJs, we analyzed NMJs by transmission electron microscopy at 12 months of age (Figure 4A), when all facets of structural degeneration mentioned previously are enhanced in Cabp7 cKO mice (Figure 3). Although the densities of mitochondria and synaptic vesicles in the presynaptic motor nerve terminals were comparable between the Cabp7 cKO and the control mice (Figures 4B and 4C), the ratio of post-versus pre-synaptic membrane length of synaptic contacts was reduced in Cabp7 cKO mice (Figure 4D), suggesting that muscle-specific deletion of Cabp7 impairs the organization and complexity of the postsynaptic membrane. Consistent with this, the size and density of junctional folds in the postsynaptic membrane of synaptic contacts were reduced in Cabp7 cKO mice, in comparison with the controls (Figures 4E and 4F). In addition, tSCs exhibited significantly enhanced penetration of their processes into the synaptic clefts, and the synaptic cleft width was increased in Cabp7 cKO mice (Figures 4G and 4H). Together, these findings indicate that muscle-specific deletion of Cabp7 induces accelerated ultrastructural degeneration of NMJs in mice at 12 months of age, in addition to the degeneration seen by optical microscopy.

### Muscle-specific deletion of Cabp7 exacerbates compound muscle action potential decrement in mice

**Figure 5:**
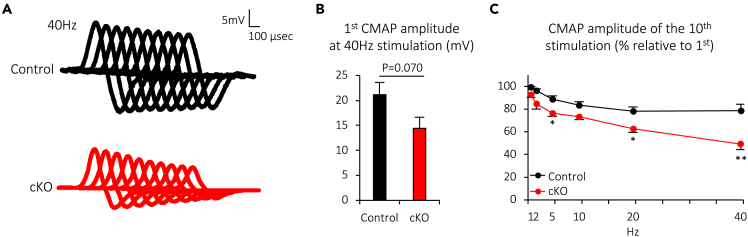
Muscle-specific deletion of Cabp7 exacerbates CMAP decrement in mice at 12 months of age (A) Representative traces of CMAP during repetitive nerve stimulation at 40 Hz in the TA muscles of Cabp7 cKO and the control mice at 12 months of age. (B and C) Quantified results for the first CMAP amplitude at 40 Hz-stimulation (B) and the amplitude ratio of the 10th to the first CMAP (C). Error bars indicate mean ± SEM (n = 5 mice for control, n = 7 mice for cKO). Asterisks denote a significant statistical difference: ∗p < 0.05, ∗∗p < 0.01 by unpaired Student’s *t* test. “N.S.” is not indicated in C for better visibility.

The aforementioned structural degeneration of NMJs accelerated by the lack of Cabp7 suggests functional deterioration of NMJs in Cabp7 cKO mice. The neurotransmitter ACh (acetylcholine) released by the motor neuron activates AChRs clustered on the postsynaptic membrane at the NMJ, which triggers generation of compound muscle action potentials (CMAPs) in mammals.^37^ The CMAP amplitude depends on the number of myofibers firing action potentials,^38^ and previous reports have shown an age-related reduction in the CMAP amplitude.^39^^,^^40^^,^^41^ Also, other studies have demonstrated an age-related exacerbation of CMAP decrement during repetitive nerve stimulation, suggesting an age-related impairment of neuromuscular transmission.^42^^,^^43^ Since age-related NMJ denervation, which inevitably impairs neuromuscular transmission, was significantly enhanced in addition to the other facets of structural degeneration of NMJs in Cabp7 cKO mice by 12 months after birth (Figures 3 and 4), we hypothesized that neuromuscular transmission and resultant firing of muscle action potentials would also be impaired. Thus, we examined CMAPs of TA muscles upon repetitive stimulation of the sciatic nerve (Figure 5). The first CMAP amplitude at 40-Hz stimulation was lower as measured, but the difference was not statistically significant (p = 0.070), in Cabp7 cKO mice as compared with the controls at 12 months of age (Figures 5A and 5B), yet the CMAP amplitude ratios of the 10th to the first trace at 5-, 20-, or 40-Hz stimulations were significantly reduced in Cabp7 cKO mice (Figure 5C), indicating a more pronounced decrement in the CMAP exhibited in Cabp7 cKO mice. Together with the structural impairment of NMJs in Cabp7 cKO mice (Figures 3 and 4), these findings indicate that muscle-specific deletion of Cabp7 in mice accelerates age-related degeneration of NMJs and exacerbates CMAP decrement over repeated stimulation likely due to impaired neuromuscular transmission, indicating that Cabp7 plays a protective role against age-related NMJ degeneration.

### Muscle-specific deletion of Cabp7 induces muscle atrophy in mice

**Figure 6:**
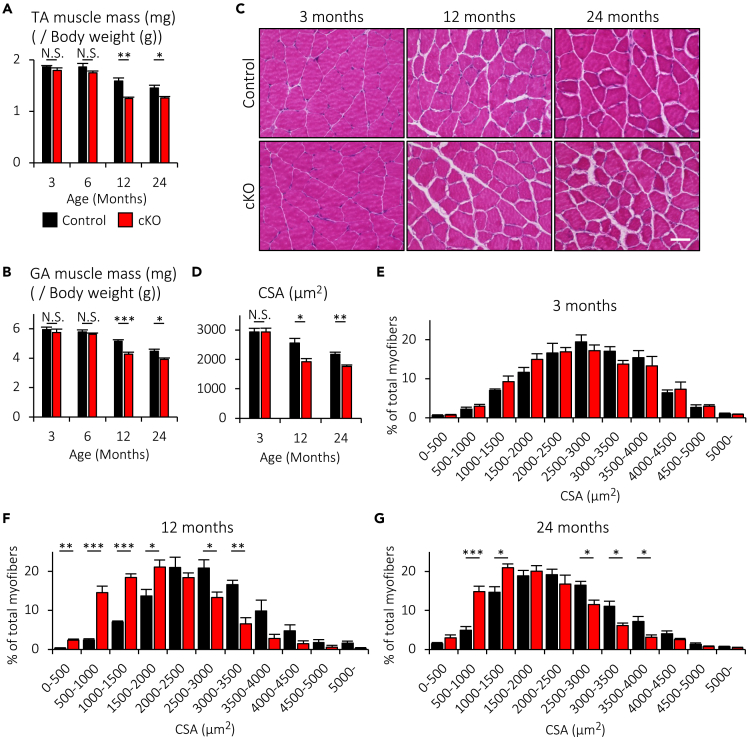
Cabp7 cKO mice show muscle atrophy (A and B) Quantification of the TA (A) and GA (B) muscle masses normalized to body weight in each mouse at the indicated ages (n = 5–8 per group). (C) Representative images of hematoxylin and eosin-stained transverse sections of the GA muscle in Cabp7 cKO and the control mice at the indicated ages. Scale bar, 50 μm. (D−G) Quantification of the myofiber cross-sectional area (CSA) of GA muscle at the indicated ages (D), and the size distribution of the CSA at 3 (E), 12 (F), and 24 (G) months of age (n = 4–5 per group). Error bars indicate mean ± SEM. Asterisks denote a significant statistical difference: ∗p < 0.05, ∗∗p < 0.01, ∗∗∗p < 0.001 by unpaired Student’s *t* test. N.S., not significant. “N.S.” is not indicated in (E−G) for better visibility. For detailed information on sample size, see Table S1.

Because impaired NMJ function may lead to muscle atrophy and weakness as observed in patients with myasthenia,^1^ we examined whether muscle-specific deletion of Cabp7 in mice affects muscle homeostasis. TA and GA muscle masses were significantly reduced in Cabp7 cKO mice at 12 and 24, but not 3 or 6, months of age, as compared with the controls (Figures 6A and 6B). Furthermore, the myofiber cross-sectional area (CSA) of GA muscles was significantly reduced in Cabp7 cKO mice at 12 and 24, but not 3, months of age (Figures 6C and 6D). In addition, Cabp7 cKO mice at 12 and 24, but not 3, months of age displayed shifts in CSA distribution with a higher frequency of small fibers in comparison with the controls (Figures 6E–6G), indicating muscle atrophy.

In general, aging is associated with a fast-to-slow muscle fiber type shift in humans and rodents, although this remains controversial.^44^^,^^45^ Thus, we examined fiber type distribution in TA muscle of Cabp7 cKO and the control mice at 12 months of age. As reported previously,^46^^,^^47^ we found that Type I slow fibers were scarce in the TA muscle (only one MyHC Type I-positive fiber in 7789 fibers of three control mice, and no MyHC Type I-positive fiber in 9130 fibers of four Cabp7 cKO mice). Furthermore, we found no significant shift of the Type II fast fiber distribution (Type IIa, Type IIx, and Type IIb) between Cabp7 cKO and the control mice (Figure S4), implying that muscle-specific deletion of Cabp7 accelerates age-related muscle fiber type shift very little, if at all. Together, these results indicate that muscle-specific deletion of Cabp7 in mice induces muscle atrophy with no obvious alteration of muscle fiber type distribution, likely due to NMJ defects, and ultimately leads to the aforementioned motor dysfunction.

### Muscle-specific deletion of Cabp7 enhances muscle expression of p25 in mice

**Figure 7:**
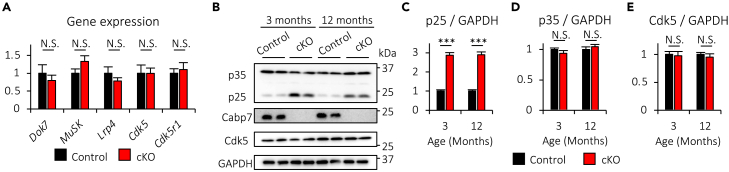
Cabp7 cKO mice show increased expression of p25 in skeletal muscle (A) Quantification of *Dok7*, *MuSK*, *Lrp4*, *Cdk5*, *Cdk5r1* mRNA expression in the TA muscle of Cabp7 cKO and the control mice at 12 months of age (n = 5 per group). The mean value of each gene expression level normalized to *Hprt* expression in the control mice was arbitrarily defined as 1. (B−E) Representative images of immunoblotting for p35/p25, Cabp7, Cdk5, and GAPDH (B) and quantification of p25 (C), p35 (D), and Cdk5 (E) expression normalized to GAPDH expression in the TA muscle of Cabp7 cKO and the control mice at 3 and 12 months of age (n = 4 per group). Error bars indicate mean ± SEM. Asterisks denote a significant statistical difference: ∗∗∗p < 0.001 by unpaired Student’s *t* test. N.S., not significant.

As mentioned, Dok-7, MuSK, and Lrp4 play essential roles in the formation and maintenance of NMJs, while Cdk5, known to be activated by p25 and the less potent activator p35, negatively regulates NMJ formation and maintenance.^1^ Thus, we hypothesized that muscle-specific deletion of Cabp7 may accelerate age-related NMJ degeneration by affecting the expression of these genes and/or proteins. We found that gene expression levels of *Dok7*, *MuSK*, *Lrp4*, *Cdk5*, and *Cdk5r1* (the gene encoding p35) in the TA muscle were comparable between Cabp7 cKO and the control mice at 12 months of age (Figure 7A), suggesting that muscle-specific deletion of Cabp7 has no significant impact on MuSK-mediated signaling, which controls *MuSK* gene expression per se.^15^^,^^48^^,^^49^ By contrast, Cabp7 cKO mice showed increased protein expression of p25, but not p35 and Cdk5, in the TA muscle at 3 and 12 months of age (Figures 7B–7E). Although p25 is generated from p35, it was reported that p25 is more stable than the short-lived protein p35,^50^ likely underlying the discrepant responses of p25 and p35 to Cabp7 deletion in the muscle. Together, our findings indicate that skeletal muscle Cabp7 suppresses muscle expression of p25 in mice.

To clarify whether muscle expression of p25, p35, and Cabp7 are altered during aging, we examined their expression in the TA muscle of WT mice at 3 and 24 months of age. The expression levels of p25 and p35 were significantly increased in the TA muscle at 24 months of age, but that of Cabp7 was not significantly altered, as compared with each value at 3 months of age (Figure S5), although the expression levels of these proteins in the control mice were comparable between 3 and 12 months of age (Figures 7B–7D). Given that muscle expression of p25, but not p35, was increased at 3 and 12 months of age in Cabp7 cKO mice (Figures 7B–7D), mechanisms underlying the enhancement of both p25 and p35 muscle expression in WT mice at 24 months of age appear to be irrelevant to the Cabp7-mediated regulation of p25 expression. Nonetheless, the increased muscle expression of p25 and p35 could contribute to age-related NMJ degeneration at least in part.

### Overexpression of p25 in skeletal muscle induces axonal swelling and nerve sprouting, and reduces the size of NMJs in mice

**Figure 8:**
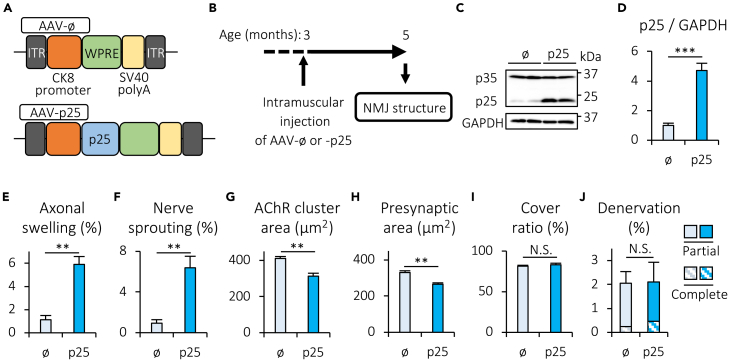
Forced expression of p25 in skeletal muscle induces NMJ degeneration (A) Diagram of AAV-ø and -p25. ITR, inverted terminal repeat; WPRE, woodchuck hepatitis virus post-transcriptional regulatory element. (B) Schematic diagram of experimental design. AAV-p25 or -ø was injected into the left hindlimb TA and GA muscles of WT mice at 3 months of age, and the morphology of NMJs was analyzed at 5 months of age (2 months after AAV injection). (C and D) Representative images of immunoblotting for p35/p25 and GAPDH (C) and quantification of p25 expression normalized to GAPDH expression (D) in the TA muscle of AAV-ø- or AAV-p25-treated mice (n = 4 per group). (E−J) Quantification of the rate of axonal swelling (E) and nerve sprouting (F), the area of postsynaptic AChR clusters (G) and presynaptic motor nerve terminals (H), the cover ratio of NMJs (I), and the rate of denervation (J) in the TA muscle of mice treated with AAV-p25 or -ø (n = 4 per group). Error bars indicate mean ± SEM. Asterisks denote a significant statistical difference: ∗∗p < 0.01, ∗∗∗p < 0.001 by unpaired Student’s *t* test. N.S., not significant.

To test the *in vivo* role of p25, whose expression is negatively regulated by Cabp7 in skeletal muscle (Figures 7B and 7C), we generated AAV-p25, a recombinant adeno-associated virus (AAV) serotype 1 vector encoding mouse p25 under the control of the muscle-specific CK8 promotor^51^ (Figure 8A). We injected AAV-p25 or empty vector (AAV-ø) into the left hindlimb TA and GA muscles of WT mice at 3 months of age (Figure 8B). Two months after the injection, we confirmed increased expression of p25 in the TA muscle of AAV-p25-treated mice, as compared with that of AAV-ø-treated mice (Figures 8C and 8D). Confocal microscopic analysis of NMJs in the TA muscle revealed that the rates of axonal swelling and nerve sprouting were increased in AAV-p25-treated mice, as compared with AAV-ø-treated mice 2 months after the injection (Figures 8E and 8F). Furthermore, the areas of AChR clusters and presynaptic motor nerve terminals were reduced in AAV-p25-treated mice in comparison with AAV-ø-treated mice (Figures 8G and 8H), whereas significant changes were not observed in the cover ratio and the denervation rate between AAV-p25- and AAV-ø-treated mice 2 months after the injection of AAV (Figures 8I and 8J). Thus, together with enhanced expression of p25 in muscle of Cabp7 cKO mice (Figures 7B and 7C), our findings indicate that muscle-specific deletion of Cabp7 induces axonal swelling and nerve sprouting, and reduces NMJ size likely, at least in part, by increasing expression of p25.

### Administration of AAV-CIP restores NMJ integrity and muscle strength, and heals muscle atrophy in Cabp7 cKO mice

**Figure 9:**
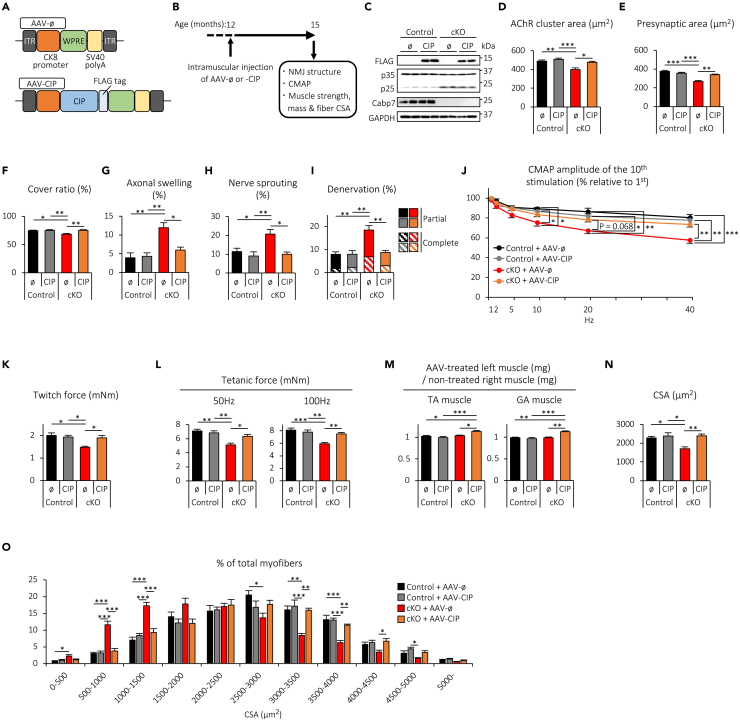
AAV-CIP administration restores NMJ integrity and muscle strength, and heals muscle atrophy in Cabp7 cKO mice (A) Diagram of AAV-ø and -CIP. (B) Schematic diagram of experimental design. AAV-CIP or -ø was injected into the left hindlimb TA and GA muscles of Cabp7 cKO and the control mice at 12 months of age. The morphology of NMJs, the CMAP, and the muscle strength, mass, and fiber CSA were analyzed at 15 months of age (3 months after AAV injection). (C) Representative images of immunoblotting for FLAG-tagged CIP, p35/p25, Cabp7, and GAPDH in the TA muscle of Cabp7 cKO or the control mice treated with AAV-CIP or -ø. (D−I) Quantification of the area of postsynaptic AChR clusters (D) and presynaptic motor nerve terminals (E), the cover ratio of NMJs (F), and the rate of axonal swelling (G), nerve sprouting (H), and denervation (I) in TA muscle (n = 5 per group). (J) Quantification of the amplitude ratio of the 10th to the first CMAP in TA muscle (n = 5–9). (K and L) Quantification of twitch (K) and tetanic force at 50 Hz- and 100 Hz-stimulations (L) (n = 5–9). (M) Quantification of the mass ratio of AAV-treated muscles to the non-treated contralateral controls of TA and GA muscles (n = 5–9). (N and O) Quantification of the CSA of GA muscle (N), and the size distribution of CSA (O) (n = 5 per group). Error bars indicate mean ± SEM. Asterisks denote a significant statistical difference: ∗p < 0.05, ∗∗p < 0.01, ∗∗∗p < 0.001 by ANOVA followed by Tukey’s *post hoc* test. “N.S.” is not indicated for better visibility. For detailed information on sample size, see Table S1.

Because p25 is a potent activator of Cdk5,^23^ our findings suggest that enhanced activity of Cdk5 may contribute to the NMJ defects caused by muscle-specific deletion of Cabp7. Previous reports showed that forced expression of CIP , a 125 amino acid peptide that is identical in sequence between mouse and human p25, inhibits p25-, but not p35-, mediated activation of Cdk5 in mouse cortical neurons and pancreatic beta cells.^29^^,^^30^ In addition, forced expression of CIP reduces Cdk5 activity and prevents neurodegenerative pathologies caused by p25 overexpression in the brain.^31^ Thus, to examine the effects of CIP expression in skeletal muscle, we generated an AAV serotype 1 vector encoding FLAG-tagged CIP under the control of the CK8 promotor (AAV-CIP) (Figure 9A) and injected AAV-CIP or AAV-ø into the left hindlimb TA and GA muscles of Cabp7 cKO and the control mice at 12 months of age (Figure 9B), when the aforementioned NMJ degeneration, including axonal swelling, nerve sprouting, and denervation, is enhanced in Cabp7 cKO mice, as compared with the control mice (Figure 3). We found that CIP expression did not affect the expression levels of p35 and p25 in the TA muscle of either Cabp7 cKO or the control mice at 15 months of age (3 months after AAV treatment) (Figure 9C), and that AAV-CIP administration in the control mice did not significantly affect the pre- and post-synaptic area, the cover ratio, axonal swelling, nerve sprouting, or denervation of NMJs (Figures 9D–9I). Furthermore, we did not observe any significant changes in the CMAP decrement of TA muscles, twitch and tetanic force of hindlimb muscles, the TA and GA muscle mass ratio to the non-treated contralateral control, or the CSA of GA muscles between AAV-CIP- and AAV-ø-treated control mice (Figures 9J‒9O). By contrast, the areas of AChR clusters and presynaptic motor nerve terminals together with the cover ratio increased in AAV-CIP-treated Cabp7 cKO mice, compared with AAV-ø-treated Cabp7 cKO mice (Figures 9D–9F). Furthermore, AAV-CIP administration in Cabp7 cKO mice significantly reduced the rates of axonal swelling, nerve sprouting, and denervation, and improved CMAP decrement (Figures 9G–9J). Note that the morphology of NMJs and the CMAP decrement in AAV-CIP-treated Cabp7 cKO mice were improved to levels similar to those seen in AAV-ø- and AAV-CIP-treated control mice (Figures 9D–9J), indicating that forced expression of CIP in skeletal muscle restores NMJ integrity in Cabp7 cKO mice.

Finally, we examined whether AAV-CIP administration affects muscle strength and mass in Cabp7 cKO mice. AAV-CIP-treated Cabp7 cKO mice showed significant increases in twitch and tetanic force of hindlimb muscles, mass ratios to the non-treated contralateral controls of TA and GA muscles, and the CSA of GA muscles, in comparison with AAV-ø-treated Cabp7 cKO mice (Figures 9K‒9O). In addition, AAV-CIP-treated Cabp7 cKO mice displayed similar levels of muscle strength and CSA to those observed in AAV-ø- or AAV-CIP-treated control mice, indicating that no muscle weakness or atrophy caused by the lack of Cabp7 remained (Figures 9K, 9L, 9N, and 9O). Together, these findings indicate that intramuscular injection of AAV-CIP restores NMJ integrity and muscle strength, and heals muscle atrophy in Cabp7 cKO mice, supporting the importance of Cabp7-mediated suppression of p25 for NMJ and muscle homeostatic resilience to aging.

## Discussion

The presynaptic motor nerve and postsynaptic muscle membrane of the NMJ undergo age-related degeneration, including increased rates of presynaptic denervation, axonal swelling, and nerve sprouting.^2^^,^^3^ Although structural degeneration does not necessarily mean functional impairment, denervation at NMJs should contribute to muscle weakness due to the loss of neuromuscular transmission. Thus, it is widely accepted that the increased rate of NMJ denervation with aging promotes age-related muscle weakness.^52^ Interestingly, it was reported that levels of MuSK activation in the muscle are reduced in aged mice as compared to those in younger ones,^43^ suggesting that impaired MuSK-mediated signaling may lead to age-related degeneration of NMJs. Consistent with this, short hairpin RNAs or Cre/loxP-mediated inactivation of MuSK in muscle leads to nerve sprouting or partial denervation at NMJs, respectively.^53^^,^^54^ Also, postnatal deletion of Lrp4 in muscle causes these types of NMJ degeneration.^55^ Furthermore, we previously demonstrated that therapeutic administration of AAV vectors encoding human Dok-7 enhances MuSK activation, NMJ innervation, and motor function in aged mice.^56^ These findings suggest that MuSK-mediated signaling has a preventive role in age-related NMJ degeneration, but its underlying mechanisms remain to be studied. In this study, we demonstrated that *Cabp7* transcripts accumulate specifically in the central region of muscle, where NMJs are formed and maintained, and that skeletal muscle-specific overexpression of the MuSK activator Dok-7 enhances the midmuscle-restricted gene expression of *Cabp7* along with that of other NMJ-related genes: *Chrna1*, *Chrne*, *AChE*, and *MuSK* (Figure 1). Also, we demonstrated that muscle-specific deletion of Cabp7 accelerated age-related degeneration of NMJs, including denervation, in mice (Figure 3). Therefore, our findings indicate that Cabp7 has an essential role in preventing age-related degeneration of NMJs, likely downstream of MuSK.

As mentioned, activation of the Ca^2+^-dependent protease calpain via cholinergic stimulation leads to the proteolytic cleavage of the Cdk5 activator p35 into the more potent activator p25, hyperactivation of Cdk5, and Cdk5-mediated dispersal of AChR clusters in cultured myotubes.^24^ However, it remained unclear whether enhanced expression of p25 in skeletal muscle affects the integrity of NMJs. Here, we demonstrated a reduction in the size of NMJs and an increase in the rate of axonal swelling and nerve sprouting in AAV-p25-treated 5-month-old mice, which showed increased expression of p25 in skeletal muscle (Figures 8C–8H). Given increased expression of p25 in skeletal muscle in Cabp7 cKO mice (Figures 7B and 7C), these findings suggest that p25 expression in skeletal muscle, normally suppressed by Cabp7, may promote degeneration of NMJs. Since p25 is produced by calpain-mediated cleavage of p35,^23^ we speculated that muscle-specific deletion of Cabp7 might enhance calpain activity by an as yet unidentified mechanism, which may cause elevated expression of p25 and subsequent NMJ defects in Cabp7 cKO mice (Figures 3, 7B, and 7C). However, AAV-p25-treated 5-month-old mice did not show any significant increase in the rate of denervation at NMJs (Figure 8J), unlike Cabp7 cKO mice (Figure 3D), suggesting that Cabp7 may play another role besides suppression of p25, or alternatively, p25-mediated denervation may require a longer time than the aforementioned, p25-mediated NMJ degeneration. It is of note that NMJ denervation in Cabp7 cKO mice was not enhanced at 3 or 6 months of age, while the size-reduction, axonal swelling and nerve sprouting of NMJs were already enhanced at 6 months of age, compared with those in the control mice (Figures 3B–3F). It should also be noted that muscle-specific deletion of Cabp7 led to structural defects not only in postsynaptic AChR clusters, but also in presynaptic motor nerve terminals and tSCs (Figures 3 and 4), suggesting that Cabp7 is involved in intercellular signaling from myotubes to motor neurons and tSCs. Therefore, elucidation of Cabp7-mediated mechanisms underlying NMJ maintenance awaits further studies.

As shown previously, intramuscular injection of AAV-CIP, which expresses the CIP derived from p25, restored NMJ integrity and muscle strength, and healed muscle atrophy in Cabp7 cKO mice (Figure 9). Given that CIP negatively regulates p25-mediated Cdk5 activation *in vitro* and *in vivo*,^29^^,^^30^^,^^31^ we hypothesized that the mechanisms underlying the beneficial effects of CIP on skeletal muscle in Cabp7 cKO mice likely involves suppression of Cdk5. Moreover, p25 also binds and activates glycogen synthase kinase 3β (GSK3β), and in silico analysis suggests that GSK3β likely interacts with the α3 and α4 helices of p25, both of which are contained within the CIP protein.^57^ Thus, forced expression of CIP in skeletal muscle might also inhibit p25-mediated GSK3β activation. Interestingly, overexpression of a constitutively active GSK3β mutant impairs AChR clustering in cultured myotubes,^58^ suggesting that GSK3β may negatively regulate the formation and maintenance of NMJs and thus might contribute to age-related degeneration of NMJs. In addition, it was interesting that AAV-CIP treatment at 12 months of age restored NMJ innervation in Cabp7 cKO mice at 15 months of age to the levels observed in the AAV-ø- or AAV-CIP-treated control mice (Figure 9I), because this suggests that intramuscular injection of AAV-CIP promotes reinnervation of denervated NMJs (see Figure 3D showing the rate of denervation in Cabp7 cKO mice at 12 months of age), in addition to the other beneficial effects on NMJs (Figures 9D–9H), in a manner dependent on the absence of Cabp7. Therefore, uncovering the molecular mechanisms underlying the effects of CIP expression on skeletal muscle in Cabp7 cKO mice would deepen our understanding of NMJ homeostasis and further provide a novel therapeutic approach for age-related NMJ defects and muscle weakness.

### Limitations of the study

Here, we identified *Cabp7* as a novel NMJ-related gene that shows midmuscle-restricted expression downstream of MuSK and further demonstrated acceleration of age-related NMJ denervation, muscle weakness and atrophy, and motor dysfunction, together with shortened lifespan in Cabp7 cKO mice that lack Cabp7 specifically in muscle. Surprisingly, intramuscular injection of AAV-CIP, expressing the CIP, restored NMJ integrity and muscle strength, and healed muscle atrophy in Cabp7 cKO mice. However, as we also demonstrated in the present study, forced expression of the potent Cdk-5 activator p25 induced no NMJ denervation for at least 2 months following intramuscular administration of AAV-p25 in mice at 3 months of age, raising the possibility that forced expression of CIP may affect as yet unidentified signaling besides that mediated by p25. Thus, developing a novel therapeutic approach aimed at restoring NMJ integrity based on our findings needs further studies, in particular asking how the AAV-CIP treatment promoted NMJ innervation.

## STAR★Methods

### Key resources table

**Table:** 

REAGENT or RESOURCE	SOURCE	IDENTIFIER
**Antibodies**		

Rabbit anti-Cabp7	This paper	N/A
Rabbit anti-GAPDH	Cell Signaling Technology	Cat# 2118; RRID:AB_561053
Mouse anti-Cdk5	Santa Cruz Biotechnology	Cat# sc-6247; RRID:AB_627241
Rabbit Anti-p35/25	Cell Signaling Technology	Cat# 2680, RRID:AB_1078214
Mouse Anti-FLAG	Merck	Cat# F3165, RRID:AB_259529
Sheep Horseradish peroxidase-labeled anti-mouse IgG	GE Healthcare	Cat# NA9310-1mL, RRID:AB_772193
Donkey Horseradish peroxidase-labeled anti-rabbit IgG	GE Healthcare	Cat# NA9340, RRID:AB_772191
Rabbit anti-neurofilament-L	Cell Signaling Technology	Cat# 2837; RRID:AB_823575
Rabbit anti-synapsin-1	Cell Signaling Technology	Cat# 5297; RRID:AB_2616578
Mouse anti-MyHC Type I	DSHB	Cat# BA-D5; RRID:AB_2235587
Mouse anti-MyHC Type IIa	DSHB	Cat# SC-71; RRID:AB_2147165
Mouse anti-MyHC Type IIb	DSHB	Cat# BF-F3; RRID:AB_2266724
Rabbit anti-Laminin	Sigma-Aldrich	Cat# L9393; RRID:AB_477163
Goat Alexa 647-conjugated anti-rabbit IgG	Thermo Fisher Scientific	Cat# A-21244; RRID:AB_2535812
Goat Alexa 647-conjugated anti-mouse IgG2b	Thermo Fisher Scientific	Cat# A-21242, RRID:AB_2535811
Goat Alexa 594-conjugated anti-mouse IgG1	Thermo Fisher Scientific	Cat# A-11032; RRID:AB_2534091
Goat Alexa 488-conjugated anti-mouse IgM	Thermo Fisher Scientific	Cat# A-21042; RRID:AB_2535711
Goat Alexa 405-conjugated anti-rabbit IgG	Thermo Fisher Scientific	Cat# A-31556; RRID:AB_221605

**Bacterial and virus strains**		

AAV1-ø	This paper	N/A
AAV1-p25	This paper	N/A
AAV1-CIP	This paper	N/A

**Chemicals, peptides, and recombinant proteins**		

Isogen II	Nippon Gene	Cat# 311-07361
GST-Cabp7	This paper	N/A
MBP-Cabp7	This paper	N/A
Glutathione Sepharose	GE Healthcare	Cat# 17513201
Amylose Resin	NEW ENGLAND Biolabs	Cat# E8021S
rProtein A-sepharose	GE Healthcare	Cat# 17127902
cOmplete protease inhibitor cocktail	Roche	Cat# 11697498001
PhosSTOP phosphatase inhibitor cocktail	Roche	Cat# 4906845001
ECL Prime Western Blotting Detection Reagent	GE Healthcare	Cat# RPN2236
CF594-conjugated α-bungarotoxin	biotium	Cat# 00007
Tissue-tek O.C.T. Compound	Sakura Finetek	Cat# 45833
Epon812	Nisshin EM	Cat# 342
Anti-Digoxigenin-AP Fab fragments	Merck	Cat# 11093274910
BCIP/NBT Color Development Substrate	Promega	Cat# S3771

**Critical commercial assays**		

RNeasy Mini Kit	Qiagen	Cat# 74104
AAVpro Titration Kit	Takara Bio	Cat# 6233
Prime Script RT Reagent Kit with gDNA Eraser	Takara Bio	Cat# RR047B
TB Green Premix Ex Taq II (Tli RNaseH Plus)	Takara Bio	Cat#RR820D

**Deposited data**		

Raw sequencing data	DDBJ	DDBJ: DRA017004

**Experimental models: Cell lines**		

HEK293EB cells	T.Matsushita et al.^59^	N/A
JM8 stem cells	EuMMCR	N/A

**Experimental models: Organisms/strains**		

C57BL/6J	Japan SLC	N/A
Dok-7 Transgenic mice	A. Inoue et al.^15^	N/A
CAG-FLPe mice	RIKEN	RRID:IMSR_RBRC01834
Cabp7^flox/flox^	This paper	N/A
HSA-Cre transgenic mice	The Jackson Laboratory	RRID:IMSR_JAX:006149

**Oligonucleotides**		

Primers for qPCR, see Table S2	This paper	N/A

**Recombinant DNA**		

pGEX-Cabp7	This paper	N/A
pMAL-Cabp7	This paper	N/A
pCK8-ø	This paper	N/A
pCK8-p25	This paper	N/A
pCK8-CIP	This paper	N/A
pRep2Cap1	Penn Vector Core	Cat# PL-T-PV0001
pHelper	Agilent Technologies	Cat# 240071
Cabp7 gene targeting vector	KOMP	Cat# PG00159_Z_8_C06

**Software and algorithms**		

cellSens Digital Imaging Software	Olympus	RRID:SCR_014551
ImageJ	NIH	RRID: SCR_003070
R	Project for Statistical Computing	RRID:SCR_001905

### Resource availability

#### Lead contact

Further information and requests for resources and reagents should be directed to and will be fulfilled by the lead contact, Yuji Yamanashi (yyamanas@ims.u-tokyo.ac.jp).

#### Materials availability

All reagents generated in this study are available from the lead contact with a completed materials transfer agreement.

#### Data and code availability

• Raw RNA sequencing data have been deposited at DDBJ (DNA DataBank of Japan) and are publicly available as of the date of publication (accession number DRA017004). All other data reported in this paper will be shared by the lead contact upon request.
• This paper does not report any original code.
• Any additional information required to reanalyze the data reported in this paper is available from the lead contact upon request.

### Experimental model and study participant details

#### Animals

All experimental procedures involving animals were approved by the Animal Ethics Committee of the Institute of Medical Science, the University of Tokyo (A21-22). Wild-type (WT) mice used in this study were on a C57BL/6J background. Dok-7 transgenic (Tg) mice, which express human Dok-7 protein tagged with Enhanced green fluorescent protein (EGFP) specifically in the skeletal muscle, have been described previously.^15^ To generate Cabp7 cKO mice, the targeting vector (Figure S1A) was obtained from the Knockout Mouse Project (KOMP) Repository and electroporated into JM8 embryonic stem (ES) cells. After selection with neomycin, positive ES clones were determined by Southern blotting (Figure S1B). The chimeras were generated by blastocyst injection of the positive ES clone, and then a germ-line chimeric male was crossed with *CAG-FLPe* females (RBRC01834, RIKEN) to generate *Cabp7*^*flox/flox*^ mice, in which two *loxP* sites were inserted into intron 1 and exon 5 of *Cabp7* gene (Figure S1C). The *Cabp7*^*flox/flox*^ mice were crossed with *HSA-Cre* transgenic mice (#006149, The Jackson Laboratory) to obtain *Cabp7*^*flox/flox*^*;HSA-Cre* male (Cabp7 cKO) mice. Age-matched *Cabp7*^*flox/+*^*;HSA-Cre* male mice were used as the control mice. All mice used in the present study were housed on a 12-h light/dark cycle under specific pathogen-free (SPF) conditions with free access to water and standard mouse chows in the animal facility of the Institute of Medical Science, the University of Tokyo. Male mice were used in the present study aside from “*Whole-mount staining of NMJs*” and “*Whole-mount in situ hybridization*”, in which embryos at E18.5 were used irrespective of sex (Figures 1D and S3).

### Method details

#### Plasmid construction

cDNA encoding the cytoplasmic region (1–188 a.a.) of mouse Cabp7 was generated by PCR for insertion into the pGEX-6P-1 and pMAL-c2X plasmids (GE Healthcare and NEW ENGLAND Biolabs), to obtain the pGEX-Cabp7 and pMAL-Cabp7 plasmids, respectively. The DNA fragments of CK8 promoter,^51^ woodchuck hepatitis virus post-transcriptional regulatory element (WPRE),^60^ and SV40 polyA signal were synthetically generated (Genscript). To obtain the pCK8-ø plasmid, the CMV promoter, beta globin intron, and *human growth hormone (hGH)* polyA signal in the pAAV-MCS plasmid (Agilent Technologies) were replaced with the CK8 promoter, WPRE, and SV40 polyA signal, respectively. cDNA encoding mouse p25^61^ or Cdk5 inhibitory peptide (CIP, a 125 amino acid peptide that is identical in sequence between mouse and human p25)^62^ fused to an FLAG tag was generated by PCR and cloned into pCK8-ø for generation of the pCK8-p25 or pCK8-CIP plasmid, respectively.

#### RNA sequencing

Total RNA was extracted from the synaptic or extrasynaptic region of diaphragm muscles (Figure 1A) in 3-month-old WT mice using RNeasy Mini Kit (Qiagen). Paired-end 125 bp RNA sequencing analyses using HiSeq2500 (Illumina) and subsequent gene expression analyses were performed by Eurofins Genomics. False discovery rate (FDR) < 0.05 was considered statistically significant (Figure 1B).

#### AAV production and administration

To generate AAV-ø, AAV-p25, or AAV-CIP, the AAV1 chimeric helper plasmid pRep2Cap1 and the adenovirus helper plasmid pHelper (Agilent Technologies) were co-transfected into HEK293EB cells^59^ with pCK8-ø, -25, or -CIP, respectively, using polyethylenimine. Five days after transfection, the culture medium was concentrated using Amicon ultra centrifugal filters (Merck Millipore) and replaced with 10 mM phosphate-buffered saline (PBS) (pH7.4). The viral titer was determined by real-time quantitative PCR using the AAVpro Titration Kit (Takara Bio), and 5 × 10^10^ vg (viral genomes) of AAV-ø or -p25 were injected into the left hindlimb tibialis anterior (TA) and gastrocnemius (GA) muscles of 3-month-old WT mice. 1.5 × 10^11^ vg of AAV-ø or AAV-CIP were administered into the left hindlimb TA and GA muscles of Cabp7 cKO or the control mice at 12 months of age. The contralateral right hindlimb was analyzed as the non-treated control. AAV administration was conducted in a blinded fashion.

#### Reverse transcription quantitative PCR (RT-qPCR)

Total RNA was extracted from the brain, the TA or GA muscles, or the whole, synaptic, or extrasynaptic region of diaphragm muscles using Isogen II (Nippon Gene) and then reverse-transcribed into cDNA using the Prime Script RT Reagent Kit with gDNA Eraser (Perfect Real Time, Takara Bio). RT-qPCR was performed using TB Green Premix Ex Taq II (Tli RNaseH Plus, Takara Bio) on a CFX ConnectTM Real-Time PCR Detection System (Bio-Rad Laboratories). Primers used for RT-qPCR are shown in Table S2.

#### Production of antibodies to Cabp7

Using pGEX-Cabp7 or pMAL-Cabp7, polypeptides of mouse Cabp7 (1–188 a.a.) fused to glutathione-*S*-transferase (GST-Cabp7) or maltose-binding protein (MBP-Cabp7) were expressed in *Escherichia coli* and purified with Glutathione Sepharose (GE Healthcare) or Amylose Resin (NEW ENGLAND Biolabs), respectively. Serum from rabbits immunized with GST-Cabp7 was subjected to ammonium sulfate precipitation, followed by incubation with rProtein A-sepharose (GE Healthcare) to purify the IgG fraction. The MBP-Cabp7-bound column was used for purification of antibodies to Cabp7.

#### Immunoblotting

Tissue lysates were prepared from the TA, GA, or diaphragm muscles, or the brain using lysis buffer containing 50 mM Tris-HCl (pH8.0), 50 mM NaCl, 1% nonidet P-40, 0.5% sodium deoxycholate, 0.001% sodium dodecyl sulfate (SDS), 1 mM EDTA, 1 mM EGTA, cOmplete protease inhibitor cocktail (Roche), and PhosSTOP phosphatase inhibitor cocktail (Roche). Proteins were separated by SDS-PAGE on 12% gels and transferred to a PVDF membrane (Merck Millipore), which was then incubated with antibodies to Cabp7, GAPDH (14C10, Cell Signaling Technology), Cdk5 (J-3, Santa Cruz Biotechnology), p35 (C64B10, Cell Signaling Technology), or FLAG (M2, Merck). The membrane was washed and then incubated with horseradish peroxidase-labeled anti-mouse or anti-rabbit IgG (GE Healthcare). The blots were visualized using an LAS4000 imager with ECL Prime Western Blotting Detection Reagent (GE Healthcare).

#### Whole-mount staining of NMJs

The TA or diaphragm muscles were fixed in 1% paraformaldehyde (PFA) in PBS and permeabilized with 1% Triton X-100 in PBS. Then, the muscles were incubated with anti-neurofilament-L (C28E10, Cell Signaling Technology) and anti-synapsin-1 (D12G5, Cell Signaling Technology) rabbit antibodies to label motor axons and presynaptic nerve terminals, respectively, followed by incubation with Alexa 647-conjugated anti-rabbit IgG and CF 594-conjugated α-bungarotoxin (biotium); the latter was used to visualize postsynaptic AChR clusters. Confocal Z serial images were collected with an FV1200 Confocal Laser Scanning Microscope (Olympus) and collapsed into a single image. The sizes (areas) of AChR clusters and presynaptic motor nerve terminals apposing AChR clusters, and the cover ratio of presynaptic motor nerve terminals to AChR clusters were quantified using cellSens Digital Imaging Software (Olympus). The NMJ was scored as complete or partial denervation when no presynaptic area was detected on the AChR cluster area^56^ or when more than 5 μm length of an AChR enriched branch within the postsynaptic area was not covered by the presynaptic motor nerve terminal,^63^ respectively. Totally or partially denervated NMJs were included in quantification of postsynaptic AChR cluster and presynaptic motor nerve terminal areas and the cover ratio. NMJs with nerve sprouting were defined by terminal extension of the nerve more than 1 μm beyond the border of AChR cluster in any direction,^64^ while those with axonal swelling were defined by bulging of the axon (more than 5 μm in diameter) proximal to the postsynaptic AChR cluster. More than 100 synaptic sites were analyzed for each mouse. These experiments were conducted in a blinded fashion.

#### Quantification of myofiber size

The GA muscles were mounted in Tissue-tek O.C.T. Compound (Sakura Finetek), and snap-frozen with liquid nitrogen. Transverse sections of GA muscle were prepared at 16 μm thickness and subjected to hematoxylin and eosin staining. Bright-field images of muscle bundles were collected with a BioREVO fluorescent microscope (Keyence). Cross-sectional area of GA muscle fiber was measured using the National Institutes of Health (NIH) ImageJ software (version 1.48v). For quantification, at least 1000 myofibers per mouse were analyzed. These experiments were conducted in a blinded fashion.

#### Myofiber typing

The TA muscles were mounted in Tissue-tek O.C.T. Compound (Sakura Finetek), snap-frozen in liquid nitrogen, and sectioned at 8 μm thickness. Then, the frozen sections were incubated with the primary antibodies specific for MyHC Type I (BA-D5), MyHC Type IIa (SC-71), MyHC Type IIb (BF-F3) (Developmental Studies Hybridoma Bank), or Laminin (L9393, Sigma), to label Type I, Type IIa, Type IIb fibers, or myofiber basal lamina, respectively, followed by incubation with isotype-specific Alexa Fluor-conjugated secondary antibodies: Alexa Fluor 647-conjugated anti-mouse IgG2b, Alexa Fluor 594-conjugated anti-mouse IgG1, Alexa Fluor 488-conjugated anti-mouse IgM, and Alexa Fluor 405-conjugated anti-rabbit IgG (Thermo-Fisher Scientific). Confocal Z serial images were collected with an FV1200 Confocal Laser Scanning Microscope (Olympus) and collapsed into a single image. For quantification, all the muscle fibers (1889–2771 fibers per mouse) were analyzed. These experiments were conducted in a blinded fashion.

#### Electron microscopy

The diaphragm muscles were dissected and fixed in 0.1% glutaraldehyde and 4% PFA in 100 mM phosphate buffer (PB) (pH 7.4) and then incubated with CF 594-conjugated α-bungarotoxin in 100 mM PB to label the AChR-rich postsynaptic membrane. The AChR-rich region of the muscles, including the presynaptic motor nerves, was dissected and refixed in 2.5% glutaraldehyde in 100 mM PB. The tissue was postfixed in 2% OsO_4_ in 100 mM PB, dehydrated, and embedded in Epon812 (Nisshin EM). The sections were cut longitudinally with 50 nm thickness, stained with uranyl acetate and Reynold’ lead citrate, and photographed in a JEM-1400Flash transmission electron microscope (JEOL Ltd.). The images were analyzed using the ImageJ software (version 1.48v).^65^ These experiments were conducted in a blinded fashion.

#### Whole-mount *in situ* hybridization

The diaphragm muscles were fixed in 4% PFA in PBS, dehydrated in methanol, digested with proteinase K, and probed with digoxigenin (DIG)-labeled antisense RNA probes corresponding to the whole coding region of mouse *Cabp7*. The muscles were washed, incubated with Anti-Digoxigenin-AP Fab fragments (Merck), and with BCIP/NBT Color Development Substrate (Promega). Photography was performed using a BioREVO fluorescent microscope (Keyence). These experiments were conducted in a blinded fashion.

#### Grip test

Forelimb grip strength was measured by a computerized electronic pull-strain gauge 1027DSM (Columbus Instruments). Mice were allowed to grasp the grid and pulled horizontally until the grip was released. Ten measurements were taken per mouse, and the average of these ten measurements was used for statistical analysis. These experiments were conducted in a blinded fashion.

#### Rotarod test

Rotarod tests were performed with an apparatus consisting of a 3.2-cm-diameter rod RRAC-3002 (O’Hara & Co.). The rotarod was set to accelerate from 2.5 to 40 rpm over a 5-min period. Before testing, each mouse was acclimated to the rotarod device for three trials per day on two consecutive days to familiarize the mice with the device and test protocols. Two measurements were taken per mouse each day, and the average of these two measurements was used for statistical analysis. These experiments were conducted in a blinded fashion.

#### Maximal plantarflexion isometric torque test

Maximal plantarflexion isometric torque was measured with PowerLab 26T data acquisition system (ADInstruments). Mice were anesthetized with isoflurane, and electrical stimulation was applied to the posterior surface of the skin of the lower limbs. To attach surface stimulation electrodes (Bio Research Center) to the skin, viscous electrically conductive gel (CR) (Sekisui Plastics) was applied between the electrodes and the skin. The electrodes were fixed with adhesive tape to the surface of the myotendinous junction and a 5-mm proximal locus. Plantarflexor muscles were percutaneously stimulated via surface stimulation electrodes, and maximal plantarflexion was evoked using a supramaximal twitch current (1-Hz frequency, 0.1-msec duration, and 8.0-mA current) and tetanic current (50- or 100-Hz frequency, 0.1-msec duration, 300-ms train duration, and 8.0-mA current). Isometric plantarflexion torque (T) was calculated from the pressure applied to a footplate (F) and the distance from the axis of the ankle joint to the sensor (r) as follows: T = Fr. These experiments were conducted in a blinded fashion.

#### Electromyography

Compound muscle action potentials (CMAPs) were studied using a PowerLab 26T data acquisition system (ADInstruments). Paired stimulating electrodes separated by 3 mm were kept in contact with the exposed sciatic nerve at 10 mm from the midline for 10 supramaximal repetitive stimulation at 1, 2, 5, 10, 20, or 40 Hz. The recording electrodes were inserted in the middle of the TA muscle whereas the reference one was inserted 5-mm distally, both of which were connected via an MPA8I preamplifier (Multi Channel Systems) to an SC8x8BC signal collector (Multi Channel Systems). To isolate stimulus artifacts, a ground electrode was placed between the stimulus and recording electrodes. Peak-peak amplitudes were determined in LabChart software (ADInstruments), and the amplitude ratio of the 10th to the first action potential was calculated. These experiments were conducted in a blinded fashion.

### Quantification and statistical analysis

Data were expressed as mean ± SEM and analyzed using Easy R software (version 4.1.1). Statistical differences were determined by two-tailed Student’s *t* test, ANOVA followed by Tukey’s *post hoc* test, or log rank test. p < 0.05 was considered statistically significant, except for RNA sequencing analysis. The sample size for statistical analyses is shown in Table S1.
