# Supplementary material for: Calcium-binding protein 7 expressed in muscle negatively regulates age-related degeneration of neuromuscular junctions in mice
Source: iScience. 2024 Jan 26;27(2):108997. doi: 10.1016/j.isci.2024.108997 (PMC10847746; doi:10.1016/j.isci.2024.108997)
Supplement: Document S1. Figures S1–S5 and Tables S1 and S2 [file mmc1.pdf]

iScience, Volume *27*

Supplemental information

Calcium-binding protein 7 expressed in muscle negatively regulates age-related degeneration of neuromuscular junctions in mice

Takahiro Eguchi, Tohru Tezuka, Yuji Watanabe, Akane Inoue-Yamauchi, Hiroshi Sagara, Manabu Ozawa, and Yuji Yamanashi

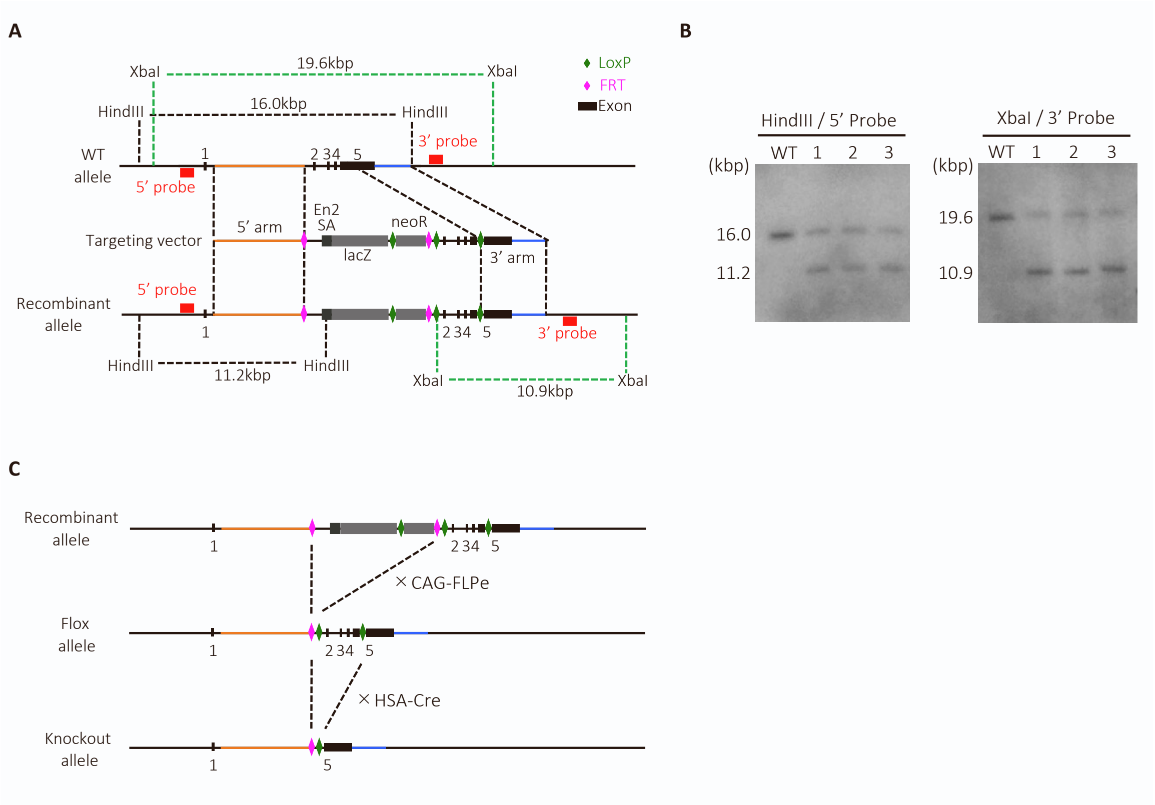

Figure S1. Generation of Cabp7 conditional knockout (cKO) mice, Related to Figure 2. (**A**) Restriction map of the WT allele, targeting vector, and the recombinant allele of the *Cabp7* gene. En2, mouse *En2* splice acceptor sequence; lacZ, *β-galactosidase* gene; neoR, *neomycin phosphotransferase* gene. (**B**) Southern blot analysis with the 5’ or 3’ probe depicted in (A) of HindIII- or XbaI-digested genomic DNA, respectively, prepared from ES clones electroporated with targeting vector (#1−3) or WT ES cells. (**C**) Schematic diagram of Cabp7 cKO mice generation. *Cabp7*$^{flox/flox}$ mice were generated by crossing mice harboring a recombinant allele with *CAG-FLPe* mice, and were crossed with *HSA-Cre* mice to generate Cabp7 cKO mice.

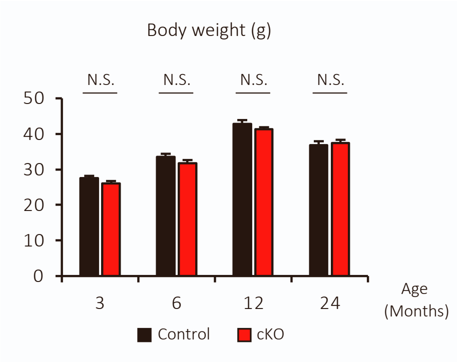

Figure S2. Muscle-specific deletion of Cabp7 does not significantly alter body weight, Related to Figure 2. Quantified results for body weights of Cabp7 cKO and the control mice at 3, 6, 12, and 24 months of age are shown. Error bars indicate mean ± SEM (n = 12−16 per group). N.S., not significant by unpaired Student's *t*-test. For detailed information on sample size, see Table S1.

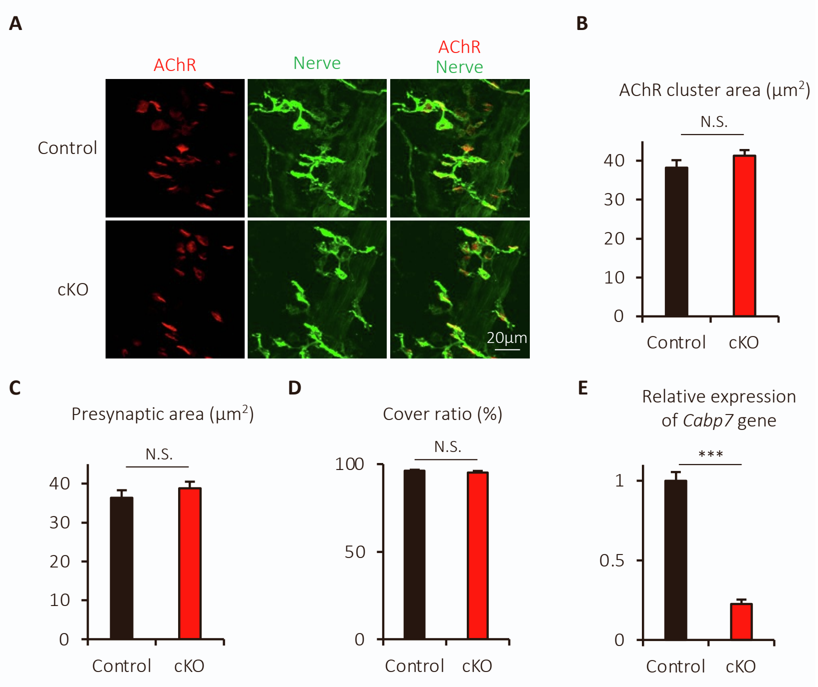

Figure S3. Muscle-specific deletion of Cabp7 does not significantly alter NMJ size at embryonic day 18.5, Related to Figure 3. (**A**) Representative images of NMJs in the diaphragm muscles of Cabp7 and the control mice at embryonic day 18.5 (E18.5). Immunohistochemistry of NMJs was performed as in Figure 3A. (**B**–**D**) Quantification of the area of postsynaptic AChR clusters (**B**) and presynaptic motor nerve terminals (**C**), and the cover ratio of NMJs (**D**). (**E**) Quantification of *Cabp7* mRNA expression in the diaphragm muscles of Cabp7 cKO and the control mice at E18.5. The mean value of *Cabp7* mRNA expression normalized to *Hprt* expression in the control mice was arbitrarily defined as 1. Error bars indicate mean ± SEM (n = 3 per group). Asterisks denote a significant statistical difference: ***P < 0.001 by unpaired Student's *t*-test. N.S., not significant.

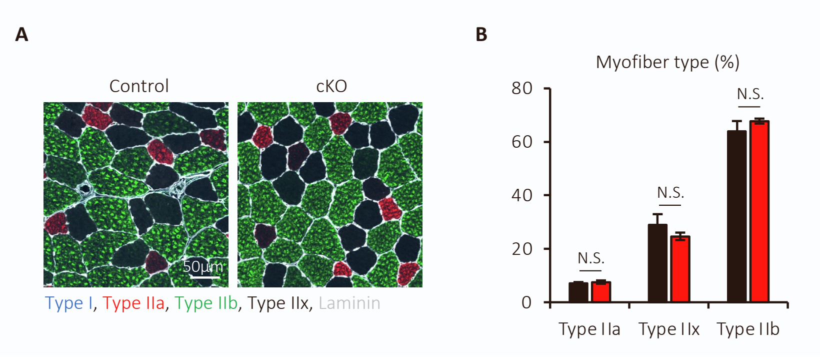

Figure S4. Muscle-specific deletion of Cabp7 does not significantly alter the distribution of myofiber type at 12 months of age, Related to Figure 6. (**A**) Representative images of immunofluorescently labeled TA muscle transverse sections of Cabp7 cKO and the control mice at 12 months of age. Type I, Type IIa, or Type IIb fibers were visualized with antibodies against MyHC Type I (blue), MyHC Type IIa (red), or MyHC Type IIb (green), respectively. Unstained fibers were counted as Type IIx fibers (black). The basal lamina was stained with laminin antibody to visualize intercellular boundaries (gray). No Type I fiber (blue) is included in these representative images (see Results for details). (**B**) Quantification of myofiber type. Error bars indicate mean ± SEM (n = 3 mice for control, n = 4 mice for cKO). N.S., not significant by unpaired Student's *t*-test.

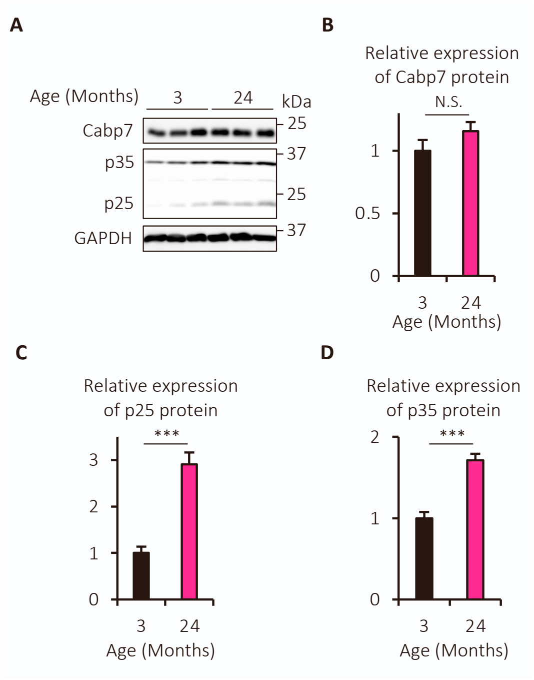

Figure S5. Muscle expression of p25 and p35, but not Cabp7, is increased at 24 months of age compared with that at 3 months of age in WT mice, Related to Figure 7. (**A**−**D**) Representative images of immunoblotting for Cabp7, p35/p25, and GAPDH (**A**) and quantification of Cabp7 (**B**), p25 (**C**), and p35 (**D**) expression normalized to GAPDH expression in the TA muscle of WT mice at 3 and 24 months of age. Error bars indicate mean ± SEM (n = 6 per group). Asterisks denote a significant statistical difference: ***P < 0.001 by unpaired Student's *t*-test. N.S., not significant.

Table S1. The sample sizes for statistical analyses in this study, Related to STAR Methods.

Fig. 1B	WT Syn	n = 3
	WT ExSyn	

Fig. 1C	WT	n = 6
	Dok-7 Tg	

	Tissues	TA	GA	Dia	Brain
Fig. 2A	Control	n = 5			
	cKO				
Fig. 2C	Control	n = 4			
	cKO				

	Age (Months)	3	6	12	24
Fig. 2D and E	Control	n = 15			n = 12
	cKO	n = 16			n = 13
Fig. 2 I-K	Control	n = 5		n = 6	n = 7
	cKO				

Fig. 2L	Control	n = 18
	cKO	n = 19

	Age (Months)	3	6	12	24
Fig. 3 B-G	Control	n = 4		n = 5	n = 6
	cKO			n = 6	

Fig. 4 B-H	Control	n = 4
	cKO	

Fig. 5B and C	Control	n = 5
	cKO	n = 7

	Age (Months)	3	6	12	24
Fig. 6A and B	Control	n = 5			n = 8
	cKO	n = 6			n = 7

	Age (Months)	3	12	24
Fig. 6 D-G	Control	n = 4		n = 5
	cKO			

Fig. 7A	Control	n = 5
	cKO	

	Age (Months)	3	12
Fig. 7 C-E	Control	n = 4	
	cKO		

Fig. 8 D-J	ø	n = 4
	p25	

	AAV	ø	CIP
Fig. 9 D-I	Control	n = 5	
	cKO		

	AAV	ø	CIP
Fig. 9 J-M	Control	n = 5	n = 6
	cKO		n = 9

	AAV	ø	CIP
Fig. 9N and O	Control	n = 5	
	cKO		

	Age (Months)	3	6	12	24
Fig. S2	Control	n = 15			n = 12
	cKO	n = 16			n = 13

Fig. S3 B-E	Control	n = 3
	cKO	n = 3

Fig. S4 B	Control	n = 3
	cKO	n = 4

Fig. S5 B-D	3 months	n = 6
	24 months	n = 6

Table S2. Primers used for quantitative RT-PCR, Related to STAR Methods.

Cabp7_Fwd	TCCCAGAGAAGTTCCATGGCACTG
Cabp7_Rev	AGTGCTCGCAGAAGGTGTCATAGAG
Dok7_Fwd	GCTTCGGCCAGTTCTCCCAGACC
Dok7_Rev	CTGTCATCCCCTCCACTGCCTG
MuSK_Fwd	CCTCAGCCCGAGATTTCTTGG
MuSK_Rev	GTCTTCCACGCTCAGAATGGT
Lrp4_Fwd	GCACACGGAATAGCCAGCA
Lrp4_Rev	GGATACAGGTACATTCGCCAAG
Cdk5_Fwd	AGGCTTCATGATGTCCTGCATAGTGAC
Cdk5_Rev	GGAATGACTTCACAATCTCAGGGTCCAG
Cdk5r1_Fwd	GTGCAGAACAGCAAGAACGCCAAG
Cdk5r1_Rev	GATGCGCGATGTTGCTCTGGTAG
Hprt_Fwd	TGGCCCTCTGTGTGCTCAA
Hprt_Rev	TGATCATTACAGTAGCTCTTCAGTCT
Chrna1_Fwd	GCTCTGTGGTGGCCATTA
Chrna1_Rev	CCGCTCTCCATGAAGTTACTCA
Chrne_Fwd	GGCAGCTTTTACCGAGAATGG
Chrne_Rev	CGGCGGATGATGAGCGTATAG
AChE_Fwd	CAAGGCCCGCTCAGGACCTG
AChE_Rev	GCCCTCGTCCTTCACCACACCCAC
